# Changes in CD4+ cells’ miRNA expression following exposure to HIV-1

**DOI:** 10.1186/1758-2652-13-S3-O11

**Published:** 2010-11-04

**Authors:** F Bignami, E Pilotti, L Bertoncelli, P Ronzi, M Gulli, N Marmiroli, G Magnani, M Pinti, C Mussini, L Lopalco, R Ruotolo, M Galli, A Cossarizza, C Casoli

**Affiliations:** 1 Università degli Studi di Milano, Dipartimento di Scienze Cliniche, Milano, Italy; 2 ANLAIDS Lombardia, Milano, Italy; 3 Università di Modena e Reggio Emilia, Dipartimento di Scienze Biomediche, Modena, Italy; 4 Università degli Studi di Parma, Dipartimento di Scienze Ambientali, Parma, Italy; 5 Arcispedale S. Maria Nuova, Unità Operattiva di Malattie Infettive, Reggio Emilia, Italy; 6 Policlinico di Modena, Clinica delle Malattie Infettive, Modena, Italy; 7 Fondazione Centro San Raffaele, Divisione di Immunologia, Malattie Infettive e Trapianti, Milano, Italy; 8 Università degli Studi di Parma, Dipartimento di Biochimica e Biologia Molecolare, Parma, Italy

## Background

Micro RNAs (miRNAs) inhibit HIV-1 expression by either modulating host innate immunity or by directly interfering with viral mRNAs. Here, we investigated the miRNA profile that discriminates different classes of HIV-1-infected patients from multiple-exposed uninfected individuals.

## Methods

The expression levels of 377 miRNAs were selectively analysed in CD4+ cells isolated from whole blood of HIV-1 élite LTNP (éLTNP), naïve, and multiply exposed uninfected individuals (MEUs). MiRNA extraction was performed by the *mir*Vana™ miRNA Isolation Kit (Ambion), and their expression was subsequently examined by real-time PCR-based arrays. The expression of miRNAs was also determined in primary culture of CD4+ T cells and monocyte-macrophages infected *in vitro* by R5 strains. Expression of Dicer and Drosha was evaluated by real-time PCR.

## Results

We only considered miRNAs that were expressed in the 70% of patients of at least one class and varied by at least one log_10_ from healthy controls. Out of 377 miRNAs, 26 were up-regulated, while 88 were down-regulated. Statistical analysis showed that 21 miRNAs significantly differentiated éLTNP from MEU and 23 miRNAs distinguished naïve from MEU, while only one (miR-155) discriminated éLTNP from naïve. By hierarchical clustering of the miRNAs according to patient class, éLTNP clustered with naïve, whereas all MEU subjects grouped together. The Dicer and Drosha expression in the patient classes correlated with miRNA profile changes. Among miRNAs differentially expressed in patient classes, 32 were detected in the *in vitro* infection model: most of the up-regulated miRNAs were expressed in monocyte-macrophages, whereas most of the down-regulated miRNAs were expressed in T lymphocytes.

## Conclusions

These findings support the consideration that the miRNA profile could be the result not only of a productive infection, but also of the exposure to HIV products that leave a signature in immune cells. These data provide some intriguing issues relative to the development of HIV vaccines targeting viral proteins.

